# Reducing Sodium Intake at the Community Level: The Sodium Reduction in Communities Program

**DOI:** 10.5888/pcd9.120081

**Published:** 2012-11-21

**Authors:** Kristy Mugavero, Jan L. Losby, Janelle P. Gunn, Jessica Lee Levings, Rashon I. Lane

**Affiliations:** Author Affiliations: Jan L. Losby, Janelle P. Gunn, Jessica Lee Levings, Rashon I. Lane, Centers for Disease Control and Prevention, Atlanta, Georgia.

Approximately 90% of Americans aged 2 years or older consume too much sodium (1). The consumption of too much sodium increases blood pressure, which increases the risk for stroke, coronary heart disease, heart failure, and renal disease (2). Population-based strategies to reduce salt intake are cost-effective, can reduce blood pressure (3), and, according to the Institute of Medicine, are needed at national, state, and community levels (2). To improve food environments and reduce sodium intake at the community level, the Centers for Disease Control and Prevention (CDC) funds the Sodium Reduction in Communities Program (SRCP). This demonstration project supports communities in creating more healthful food environments and aims to expand the evidence base for effective community strategies to address sodium intake at the population level. In this article, we describe the role of communities and environments in influencing health and strategies being implemented and evaluated by SRCP communities.

## Making the Case for Community-Level Action

Local efforts can improve local nutrition environments (4) and inform national action in support of more healthful environments (5). SRCP builds on successful examples of community-level action such as menu labeling and prevention and control of tobacco use. State and local menu labeling laws helped influence the passage of a federal menu labeling law that will make information available for consumers nationwide (5). Similarly, tobacco use prevention and control efforts at the state and local levels contributed to dissemination and implementation of successful public health strategies across the country (5).

Environment influences food choice and behavior (2,4,5), and healthful eating is possible when nutritious options are available and accessible (4). Most of the sodium Americans consume is already in the foods they purchase (ie, restaurant and processed foods) (2). Increasing access to and availability of more healthful items and providing access to information about sodium content in products may give consumers greater control over their sodium intake. Although states and communities have a role in improving food environments (2,5), information at the community level is still needed about which strategies most effectively improve availability and access to healthful foods (4) and how to implement interventions to reduce population-level sodium intake (3).

## Sodium Reduction Strategies in Communities

In September 2010, CDC awarded 6 communities (Table) funding to promote and implement strategies and conduct program evaluations through local venues to expand the evidence base on sodium-reduction strategies. (6). As a demonstration project, a key aspect of SRCP is monitoring and documenting how communities are adopting and implementing sodium-reduction strategies and the extent to which these strategies translate into changes in the food environment.

**Table:** Five Sites Awarded CDC Funding in 2010 to Implement Strategies for Reducing Sodium Intake Through Local Venues

Venue	Community				

	California^a^	Kansas^b^	Los Angeles County^c^	New York City^d^	New York State^e^
Government agencies	×	×	×		
Grocery/corner stores		×			×
Hospitals			×	×	
Private businesses		×			
Restaurants	×			×	×
Schools	×		×		×
Senior meal programs					×

Abbreviation: CDC, Centers for Disease Control and Prevention.

^a^ Grantee was Shasta County (http://healthyshasta.org/saltsavvy).

^b^ Grantee was Shawnee County (http://spotthesalt.com).

^c^ Grantee was Los Angeles County (http://www.choosehealthla.com/eat-healthy/salt/).

^d^ Grantee was New York City (http://www.nyc.gov/html/doh/html/cardio/cardio-salt-initiative.shtml).

^e^ Grantees were Broome County (http://www.gobroomecounty.com/hd/broome-county-announces-national-sodium-reduction-initiative-grant-award) and Schenectady County (http://www.schenectadycounty.com/FullStory.aspx?m=853&amid=9803).

The strategies can be grouped into 4 general approaches: 1) procurement, 2) reformulation, 3) product placement, and 4) consumer awareness. These strategies are not mutually exclusive, and communities are finding that a combination of strategies is often most effective.

### Procurement

Organizations that purchase and provide meals — such as local and state governments, programs that provide foods to citizens, private businesses, schools, hospitals, and other institutions — can influence the food environment by adopting policies that require that the food they purchase, provide, or make available contains key nutrients at levels that meet standards and that adhere to dietary guidelines (7). In an effort to improve the nutritional quality of food and beverages sold in county facilities and programs, and in part because of increased awareness of the dangers of excess sodium intake through SRCP, the County of Los Angeles Board of Supervisors in 2011 passed a motion granting the public health department authority to review and make recommendations for any request for proposals for county food services contracts to ensure that dietary requirements in the final contract promote healthy nutrition, including sodium reduction (8). Within a year of passage, the County of Los Angeles Department of Public Health has worked with the Department of Public Works, the Probations Department, and the Department of Health Services to institute changes to improve nutrition and increase access to and promotion of lower-sodium food items within county facilities and programs. Both Shawnee County, Kansas, and Shasta County, California, are exploring how procurement standards could be introduced for vending machines in city or county facilities that provide snacks to employees and visitors (6).

In the absence of a city- or county-level policy, local health departments and other government and nongovernment agencies can incorporate contractual stipulations to increase the healthfulness of foods and model more healthful food environments. Broome County, New York, issued a request for proposals for vending equipment and services that included nutritional requirements for foods and beverages sold in county government buildings (9).

Another purchasing action to reduce sodium intake is to increase buying power by combining resources. Schenectady County, New York, is forging partnerships with local independent restaurant owners and the founder of a restaurant cooperative to assess the effectiveness of leveraging multiple buyers to increase demand for lower-sodium ingredients. Shasta County is working with multiple school districts to increase participation in a local cooperative to leverage buying power to increase demand and affordability of lower-sodium items.

### Reformulation

Because most of the sodium Americans consume is found in restaurant and processed food (2), several SRCP communities are working with venues to modify cooking practices and limit the use of processed foods. Shasta County is working with schools to reduce the amount of sodium students consume in schools by increasing from-scratch cooking and using a steam table for serving food; its goal is to increase the number of fruit and vegetable offerings. Schenectady County is working with partners to change products and modify recipes for meals served at county senior meal sites and through home-delivered meal programs. These changes have resulted in an almost 10% average sodium reduction across a 5-week rotating menu, and preliminary evaluation data suggest that meal participants are accepting of the changes (10).

### Product placement

Communities are working in retail settings to make changes in product placement to make healthful choices more easily identifiable and accessible. New York City is working with hospitals to promote healthful food and sodium reduction by developing and implementing hospital retail standards, which include standards for food items as well as product promotion, placement, and portion size (11). Shawnee County is working with local convenience stores to explore ways to place more healthful food options into 1 area of each store so consumers can more easily identify and purchase them (6).

### Consumer awareness

Increasing consumer awareness is vital for increasing demand for greater choice and for ensuring successful sodium-reduction strategies (2). Consumers should be made aware of the relationship between sodium intake and blood pressure and the primary sources of sodium: restaurant and processed foods. Each SRCP community is implementing a communications component to augment other strategies for improving food environments, and each is evaluating this component to assess the effect on consumer knowledge, attitudes, and behaviors associated with sodium consumption.

Readily available information about the nutritional content of foods can assist consumers in making more healthful choices, and communities can implement innovative strategies for providing this information. Shasta County is working with independent restaurants to implement a restaurant recognition program that allows participating restaurants to identify menu items that meet defined nutritional standards, including those related to sodium, at the point of purchase (12).

## Evaluation of Community Efforts

A key component of SRCP is evaluating the effectiveness of strategies being implemented. The evaluation consists of a 3-year cross-site process and outcome case study for each community by an outside evaluator and a local evaluation conducted by each community; results will be available in 2013. As communities conduct evaluation to build the evidence base around sodium reduction strategies, early results indicate that communities are making progress toward meeting SRCP’s short-term outcome of facilitating environmental changes that increase availability of and access to lower sodium foods and the long-term goal of reducing sodium intake to recommended limits. 

## Conclusion

Communities can lead in adopting population-level approaches to reducing sodium consumption, a key strategy for reducing blood pressure levels. The SRCP supports communities in implementing and evaluating strategies related to food procurement, food reformulation, placement strategies, and consumer awareness. This work will provide insight into community-level strategies that can successfully create more healthful food environments and reduce sodium consumption, contributing to the larger goal of reducing cardiovascular disease.

## References

[1] Centers for Disease Control and Prevention. Vital signs: food categories contributing the most to sodium consumption — United States, 2007–2008. MMWR Morb Mortal Wkly Rep 2012;60(Early Release):1–7. 22318472

[2] Institute of Medicine. Strategies to reduce sodium intake in the United States. Washington (DC): The National Academies Press; 2010.

[3] Wang G , Labarthe D . The cost-effectiveness of interventions designed to reduce sodium intake. J Hypertens 2011;29(9):1693–9. 10.1097/HJH.0b013e328349ba18 21785366 PMC4544735

[4] Story M , Kaphingst KM . Robinson, O’Brien R, Glanz K. Creating healthy food environments. Annu Rev Public Health 2008;29:253–72. 10.1146/annurev.publhealth.29.020907.090926 18031223

[5] Graff SK , Kappagoda M , Wooten HM , McGowan AK , Ashe M . Policies for healthier communities: historical, legal, and practical elements of the obesity prevention movement. Annu Rev Public Health 2012;33:307–24. 10.1146/annurev-publhealth-031811-124608 22224883

[6] US Department of Health and Human Services, Centers for Disease Control and Prevention, Division for Heart Disease and Stroke Prevention. Sodium reduction in communities. http://www.cdc.gov/dhdsp/programs/sodium_reduction.htm. Accessed April 20, 2012.

[7] Improving the food environment through nutrition standards: a guide for government procurement. Washington (DC): US Department of Health and Human Services, Centers for Disease Control and Prevention, National Center for Chronic Disease Prevention and Health Promotion, Division for Heart Disease and Stroke Prevention; 2011.

[8] Strategic Alliance. ENACT local policy database. http://eatbettermovemore.org/sa/policies/policy_detail.php?s_Search=&issue=&env=&keyword=107&s_State=&jurisdiction=&year=&Search_PolicyPage=2&policyID=398. Accessed April 20, 2012.

[9] Broome County Division of Purchasing request for proposal for vending equipment and services for various Broome County departments. Broome County Strategic Alliance for Health; 2012. http://www.gobroomecounty.com/files/hd/steps/pdfs/RFP_VENDING_MACHINE_SERVICES.pdf. Accessed June 21, 2012.

[10] Public health practice stories from the field: Schenectady County program lowers sodium in menu items for seniors. Centers for Disease Control and Prevention. http://www.cdc.gov/stltpublichealth/phpracticestories/pdfs/PHPSFF_Schenectady_v2.pdf. Accessed April 20, 2012.

[11] Healthy hospital food initiative. New York City Department of Health and Mental Hygiene; 2012. http://www.nyc.gov/html/doh/html/cardio/cardio-hospital-food-initiative.shtml. Accessed June 21, 2012.

[12] Healthy kids choice. Healthy Shasta. 2011. http://healthyshasta.org/healthykidschoice.htm . Accessed June 21, 2012.

